# Supplementary material for: From sequence to enzyme mechanism using multi-label machine learning
Source: BMC Bioinformatics. 2014 May 19;15:150. doi: 10.1186/1471-2105-15-150 (PMC4229970; doi:10.1186/1471-2105-15-150)
Supplement: Additional file 2 — Java code of ml2db. Additional file ml2db_code.tar.gz contains the Java source code to run the multi-label machine learning experiments and save the results to database. The code’s Javadoc is included. [file 1471-2105-15-150-S2.zip › additional file 2/ml2db/utils/doc/uk/ac/ed/inf/utils/collectionutils/VectorUtils.html]

VectorUtils


---


|  |  |  |
| --- | --- | --- |
| **Overview** \| **Package** \| **Class** \| **Use** \| **Tree** \| **Deprecated** \| **Index** \| **Help** | |  |
| **PREV CLASS**   NEXT CLASS | **FRAMES**    **NO FRAMES**     **All Classes** |  |
| SUMMARY: NESTED \| FIELD \| CONSTR \| METHOD | DETAIL: FIELD \| CONSTR \| METHOD |  |


---


## uk.ac.ed.inf.utils.collectionutils Class VectorUtils

```
java.lang.Object
  uk.ac.ed.inf.utils.collectionutils.VectorUtils
```

---

``` public class VectorUtils extends java.lang.Object ```

---

**Constructor Summary**

`VectorUtils()`


| **Method Summary** | |
| --- | --- |
| `static java.util.ArrayList<java.lang.String>` | `listFromVector(java.util.Vector<java.lang.String> vector)` |
| `static java.util.Vector<java.lang.String>` | `trimList(java.util.Vector<java.lang.String> list)`             trim all trailing white spaces from strings in a list (beware: null elements become empty strings) |

**Methods inherited from class java.lang.Object**

`equals, getClass, hashCode, notify, notifyAll, toString, wait, wait, wait`

**Constructor Detail**

### VectorUtils

```
public VectorUtils()
```


**Method Detail**

### listFromVector

```
public static java.util.ArrayList<java.lang.String> listFromVector(java.util.Vector<java.lang.String> vector)
```

---


### trimList

```
public static java.util.Vector<java.lang.String> trimList(java.util.Vector<java.lang.String> list)
```

:   trim all trailing white spaces from strings in a list (beware: null
    elements become empty strings)

    :   **Parameters:**: `list` - **Returns:**: a list of trimmed strings


---


|  |  |  |
| --- | --- | --- |
| **Overview** \| **Package** \| **Class** \| **Use** \| **Tree** \| **Deprecated** \| **Index** \| **Help** | |  |
| **PREV CLASS**   NEXT CLASS | **FRAMES**    **NO FRAMES**     **All Classes** |  |
| SUMMARY: NESTED \| FIELD \| CONSTR \| METHOD | DETAIL: FIELD \| CONSTR \| METHOD |  |


---
